# Integrated Analyses of Mouse Stem Cell Transcriptomes Provide Clues for Stem Cell Maintenance and Transdifferentiation

**DOI:** 10.3389/fgene.2020.563798

**Published:** 2020-09-04

**Authors:** Li-Juan Wang, Xiao-Xiao Li, Jie Hou, Xin-Hua Song, Wen-Hai Xie, Liang Shen

**Affiliations:** ^1^Zibo Key Laboratory of New Drug Development of Neurodegenerative Diseases, Shandong Provincial Research Center for Bioinformatics Engineering and Technique, Institute of Biomedical Research, Shandong University of Technology, Zibo, China; ^2^School of Life Sciences, Shandong University of Technology, Zibo, China

**Keywords:** stem cell, transcriptomes, transdifferentiation, co-expression, network

## Abstract

*In vivo* cell fate reprogramming has emerged as a new method for understanding cell plasticity and as potential treatment for tissue regeneration. Highly efficient and precise reprogramming requires fully understanding of the transcriptomes which function within different cell types. Here, we adopt weighted gene co-expression network analysis (WGCNA) to explore the molecular mechanisms of self-renewal in several well-known stem cell types, including embryonic stem cells (ESC), primordial germ cells (PGC), spermatogonia stem cells (SSC), neural stem cells (NSC), mesenchymal stem cells (MSC), and hematopoietic stem cells (HSC). We identified 37 core genes that were up-regulated in all of the stem cell types examined, as well as stem cell correlated gene co-expression networks. The validation of the co-expression genes revealed a continued protein-protein interaction network that included 823 nodes and 3113 edges. Based on the topology, we identified six densely connected regions within the continued protein-protein interaction network. The SSC specific genes *Itgam*, *Cxcr6*, and *Agtr2* bridged four densely connected regions that consisted primarily of HSC-, NSC-, and MSC-correlated genes. The expression levels of identified stem cell related transcription factors were confirmed consistent with bioinformatics prediction in ESCs and NSCs by qPCR. Exploring the mechanisms underlying adult stem cell self-renewal will aid in the understanding of stem cell pool maintenance and will promote more accurate and efficient strategies for tissue regeneration and repair.

## Introduction

In mammals, different tissues and organs exhibit different capacities for regeneration that are based on the regenerative capacities of endogenous stem cells (Xia et al., 2018). ESCs are pluripotent stem cells that are able to differentiate into more than 220 cell types within the adult human body (Thomson et al., 1998). Since the initial isolation of embryonic stem cells (ESCs), stem cell biology has attracted increasing attention. Although the capacity of tissue regeneration in mammals is progressively lost during development, several tissues and organs still maintain stem cell niches that produce progeny cells which promote tissue regeneration (Adams and Scadden, 2006; Oatley et al., 2009). Accumulating evidence also indicates that the isolation of stem cells from others tissue or organs, including the intestine, liver, and teeth, is feasible and that those stem cells can maintain the regeneration of their respective tissues under certain circumstances (Hosoya et al., 2020; Takahashi and Shiraishi, 2020). The best-characterized types of adult stem cells in mammals are hematopoietic (HSCs), neural (NSCs), mesenchymal (MSCs), and spermatogonia (SSCs) stem cells. HSCs, NSCs, and SSCs can be respectively differentiated into special lineage progeny cells such as bloods cells, neurons or glia cells, and spermatogonia. MSCs are multipotent adult stem cells that can differentiate into many kinds of tissues that include bone, cartilage, muscle, fat cells, and connective tissue (Prockop, 1997). Based on their diversity, multipotency and availability, MSCs have successfully attracted increasing attention for use in tissue repair and regeneration (Nimiritsky et al., 2019; Yin et al., 2019).

Certain tissue-derived stem cells can not only differentiate into destined progeny cells but also transdifferentiate into different stem cell lineages. ESC and MSC-derived neural precursors and neurons have been used in cell therapy strategies to cure neurodegenerative diseases (Lo Furno et al., 2018; Zhang et al., 2020). SSCs that are unipotent in regard to spermatogenesis have also been reprogrammed into pluripotent stem cells (Guan et al., 2006). Induced pluripotent stem cells (iPS) that were generated directly from fibroblasts have clarified the mechanisms underlying transdifferentiation and can provide an endless supply of cells for patient-specific or disease-specific cell therapy without the issue of ethical controversy (Takahashi and Yamanaka, 2006; Takahashi et al., 2007). As the transfection of inducing transcription factors typically requires the use of a retrovirus or lentivirus vector, an elevated risk for the formation of teratomas exists in iPS cell-based therapy. Non-integrating techniques for the generation of iPS have become available in recent years, and these include the use of mRNA (Sridhar et al., 2016), proteins (Nordin et al., 2017), and small chemical molecules (Hou et al., 2013). The risk of teratoma formation also exists when non-integrating techniques are used to generate iPS, and this risk is primarily due to the remainder undifferentiated pluripotent cells after transplantation (Knoepfler, 2009). Direct transdifferentiation, which involves the conversion of somatic cells into other terminal differentiated cell lineages, offers a safer and more attractive strategy for tissue repair (Gascon et al., 2017; Ghiroldi et al., 2017; Pereira et al., 2019). The exogenous expression of neural lineage-specific transcription factors *Ascl1*, *Brn2*, and *Myt1l* successful convert fibroblasts into neurons directly (Caiazzo et al., 2015). The use of another five-factors cocktail that included *Foxg1*, *Sox2*, *Ascl1*, *Dlx5*, and *Lhx6* also converted fibroblasts into induced GABAergic interneurons (Colasante et al., 2015). Human gingival fibroblasts could be induced to transdifferentiate into functional osteoblasts via epigenetic modification and the induction of osteogenic signaling *in vitro* and *in vivo* (Cho et al., 2017). Several pancreatic transcription factors have been found to induce liver transdifferentiation into pancreatic tissues (Luo et al., 2014). Small molecules could also convert fibroblasts into islet-like cells by allowing these cells to avoid a pluripotent state (Li et al., 2014). Studies focusing on liver regeneration have suggested endogenous reprogramming as a therapeutic strategy for cell repair. Ectopic expression of *Foxa3*, *Gata4*, *Hnf1a*, and *Hnf4a* can convert murine myofibroblasts into hepatocyte-like cells *in vivo* (Song et al., 2016; Cheng et al., 2019). Ectopic expression of *Sox2* is sufficient to convert astrocytes into ASCL1-positive neural progenitors (Niu et al., 2013). Further studies revealed that *Sox2*-dependent *in vivo* reprogramming is regulated by several transcription factors, such as *Ascl1*, *Nr2e1*, *p53* and *p21* (Islam et al., 2015; Wang et al., 2016). Brain glial cells can also be directly reprogrammed into neurons by ectopic expression of other transcription factors, such as *Brn2*, *Myt1l*, *Neurod1*, and *Neurog2* (Grande et al., 2013; Torper et al., 2013; Guo et al., 2014). *In vivo* cell fate reprogramming has emerged as a new means of understanding cell plasticity and as a potential treatment in tissue regeneration. A more comprehensive understanding of the mechanisms underlying cell fate reprogramming would promote the development of more accurate and efficient strategies for tissue regeneration and repair.

Based on the observation that terminally differentiated cells can be reprogrammed into different cell lineages, transdifferentiation of stem cells may also be feasible. Understanding mechanisms govern the special properties of stem cells will provide useful information for tissue repair and regeneration. To develop safety strategies for stem cell transdifferentiation *in vivo*, it is necessary to clarify the mechanisms underlying the regulation and function of different stem cell types. Comprehensive comparisons of transcriptome characteristics of adult stem cells remain relatively rare. Here, we focus on the underlying molecular mechanisms governing self-renewal in several well-known stem cell types. WGCNA can be used to identify co-expression networks associated with different cell types (Langfelder and Horvath, 2008; Yandim and Karakulah, 2019; Yang et al., 2019; Liao et al., 2020). The expression profiles of the six best-characterized types of stem cells (ESCs, HSCs, MSCs, SSCs, PGCs, and NSCs) were re-analyzed by WGCNA. Six stem cell correlated gene co-expression networks in conjunction with identified transcription factors were constructed, and 37 genes were identified as up-regulated genes in all of these stem cells. The validation of the co-expression gene networks resulted in construction of a continued protein-protein interaction network for stem cell transdifferentiation. The topology analysis of the continued protein-protein interaction network provided useful information in regard to stem cell maintenance and transdifferentiation. CTCF binding motif analyses indicated that stem cells occupy less CTCF, indicating that stem cells require chromosome opening activity to maintain stem cell-specific stemness properties. Clarifying the mechanisms underlying stem cell self-renewal will aid us in understanding of this process and will allow us to develop more accurate and efficient strategies for tissue regeneration and repair.

## Materials and Methods

### Transcriptional Profiling of Stem Cells

The gene expression profiles of ESCs (Zhao et al., 2009), PGCs (Miyoshi et al., 2016), SSCs (Oatley et al., 2009), NSCs (Azim et al., 2015), MSCs (Wislet-Gendebien et al., 2012), and HSCs (Kvinlaug et al., 2011) were downloaded from public database (Gene Expression Omnibus database, GEO). In order to obtain the stem cell up-regulated genes, mouse embryo fibroblast cells (MEF) were used as a control to remove genes that are common between stem cells and MEF. All datasets were hybridized on the Mouse Genome 430 2.0 array. The Affy package of R was used to process the initial datasets (Gautier et al., 2004). The RMA function was adopted to obtain the expression of probes. PCA of the datasets displayed different developmental potentials among the selected stem cell types. Respective stem cell correlated gene lists were obtained by comparing the expression profiles to those of MEFs based on linear models and empirical Bayes methods. Benjamini and Hochberg’s method was used to control for the false discovery rate (FDR), and we used the adjusted *p*-values to control for the FDR. The FDR ratio was set as 5%. Adjusted *p*-values of <0.01 and log (Foldchange) of >1.5 were selected as the thresholds for the stem cell up-regulated genes. The UpSetR package was used to display the interactions among the stem cell up-regulated genes for all six of the stem cell types (Conway et al., 2017).

### Weighted Gene Co-expression Network Analysis

The respective stem cell correlated genes were combined to form an expression matrix that was used for further research. WGCNA was employed to identify co-expression modules (Langfelder and Horvath, 2008). The power of 20 was adopted as soft-threshold for the analysis. Depending upon the resulting adjacency matrix, we calculated the topological overlap matrix to measure the interconnectedness of the co-expression network. The Dynamic Hybrid Tree Cut algorithm was used to define modules that were representative of the co-expression genes. Modules with a correlation of above 0.85 were merged together. After identifying the co-expression modules, we associated these modules with respective cell types. Modules with a correlation of >0.80 (*p* < 0.01) were designated as stem cell correlated modules.

### Topological Analysis of Modules That Are Associated With Stem Cell Types

The genes within stem cell correlated modules that exhibit high connectivity may play similar roles in the maintenance of stem cell properties. Cytoscape (version 3.7.1) software was used to performed topological analysis of the co-expression network (Shannon et al., 2003). Hub genes were identified based on a high degree of connectivity among the genes in the respective modules. The Cytoscape plug-in MCODE was used to identify densely connected regions from the entire network (Bader and Hogue, 2003). We selected clusters exhibiting MCODE scores of >3.5, and greater than 10 nodes were used as candidate clusters. Transcription factor annotation was performed based on AnimalTFDB 3.0 (Hu et al., 2018).

### Quantitative RT-PCR

Neural stem cells were isolated from E12.5 mouse embryonic cortex conserved in our lab. ESCs and MEF were gifts from Kang Zou. Total RNA from ESCs, NSCs and MEF were extracted using Trizol reagent (Invitrogen). Primescript Reverse Transcriptase (Thermo Fisher) was used to synthesize cDNA. The detection of genes expression was performed based on Real-time PCR with SYBR Green (BioTeke). The expression levels of all the genes tested were determined relative to β-actin transcript levels. Primer sequences are listed in Supplmentary Table S1.

### Gene Ontology Analysis

Gene ontology analyses of identified modules and core genes were performed by DAVID (Huang da et al., 2009). The corrected FDR with a *p* value of <0.05 was selected as threshold for enriched GO terms.

### CTCF Analysis of Respective Stem Cell-Specific Hub Genes

CTCF binding motif analysis was performed using CTCFBSDB 2.0 which was developed for CTCF binding sites and genome organization (Ziebarth et al., 2013). The top 20 hub genes within the respective stem cell-specific modules were analyzed for the presence of a CTCF binding motif. Random sampling was used to evaluate the genome wide CTCF binding sites. Random samplings of 20 genes were performed on the formed expression matrix. Random sampling was performed 100,000 times to reflect the random occupancy rates of the CTCF binding motif.

### Cross-Species Annotation Between Mouse and Human Stem Cells Up-Regulated Genes

Human ESC, NSC, SSC, MSC, HSC, and fibroblast datasets that based on the microarray platform GPL570 from GEO database. Fibroblast datasets were used as a control to obtain human stem cell up-regulated genes. The parameters of human stem cell data analysis are the same as used in mouse data mining. The R package of homologene was used to cross-species annotation.

## Results

### Transcription Profiles of Respective Stem Cell Types

**FIGURE 1:**
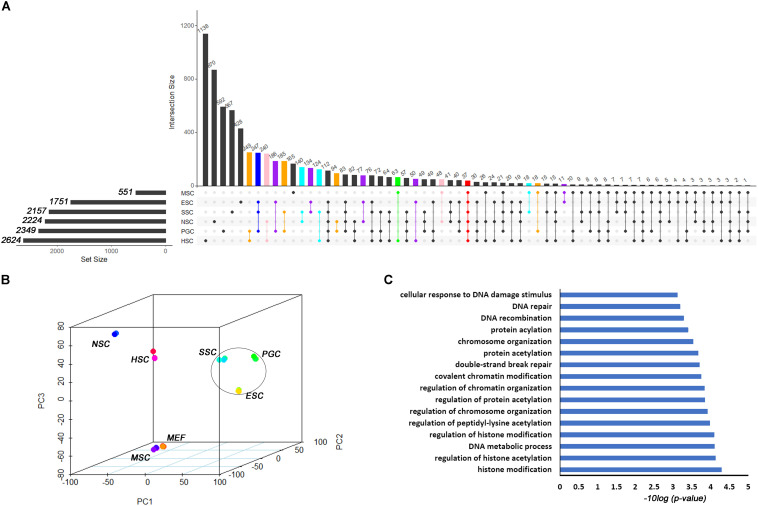
Transcription profiles for ESCs, PGCs, SSCs, NSCs, MSCs, and HSCs. **(A)** The intersection of highly expressed genes from the six types of stem cells. Dots represents the intersections of the six types of stem cells in the Venn analysis. The bar represents the size of the individual intersections. Dots with lines show highly expressed genes shared by the lined stem cells types. Different intersections of those six types of stem cells are highlighted with different colors. The 37 commonly highly expressed genes among the six types of stem cells are highlighted with red bar. **(B)** PCA revealed differences among the six types of stem cells. **(C)** GO analysis of the biological function of the core 37 core commonly highly expressed genes. The benjamini-adjusted *p* value of <0.05 was selected as threshold for enrichment of GO_BP_FAT terms.

Based on the different expression analysis, we obtained a list of genes that were up-regulated in specific stem cell types that included ESCs (1751), PGCs (2349), SSCs (2157), NSCs (2224), MSCs (551), and HSCs (2624) (Figure 1A). We also observed 64 intersections among these highly expressed specific stem cell genes. The highly expressed gene lists for each stem cell type are provided in Supplementary Table S2. The number of genes only in the respective stem cell was lower than 40%, in contrast to up-regulated genes (Supplementary Figure S1). The ESCs-only section was 24.4%. The proportions of ESCs, PGCs and SSCs were more than the other three adult stem cells. ESCs, PGCs, and SSCs shared 247 genes, as shown with a blue bar in Figure 1A; this was consistent with the pluripotency of ESCs, PGCs, and SSCs. Several studies have demonstrated that PGCs and SSCs possess a pluripotency similar to ESCs under proper conditions (Matsui et al., 1992; Kanatsu-Shinohara et al., 2004; Seandel et al., 2007). Principal Component Analysis (PCA) revealed that ESCs, SSCs, and PGCs were more similar to each other than the other three types of stem cells (Figure 1B). Bone-derived MSCs was more related to mouse embryo fibroblast cells (MEF) than HSCs. The highest ratio of those six stem types was HSCs, which was coincident with the unipotent stem cell of HSCs. The intersect sizes of ESCs and the other five types of stem cells were 186 (PGC), 134 (SSC), 77 (NSC), 10 (MSC), and 50 (HSC), which are shown with the purple bar in Figure 1A. The Venn analysis revealed that the six stem cell types shared 37 common genes (Figure 1A). The relative expression values of those 37 core genes among stem cells and MEF are shown in Supplementary Figure S2. The clustering of the stem cell types was consistent with the result of PCA. ESCs, SSCs, and PGCs were more closely to each other. The 37 core genes were assigned to two large blocks based on heatmap analysis (Supplementary Figure S2). *Cdca5*, *Trim37*, *Mllt4*, and other 15 other genes were highly expressed compared to MEF. The expressions of the 37 highly expressed core genes are detailed in Table 1. The foldchanges of the respective types of stem cells were compared to MEF. The Gene Ontology (GO) analysis of the biological function of the 37 common up-regulated genes indicated that these genes were involved in DNA recombination, chromatin organization, and DNA metabolic processes (Figure 1C). *Trim37*, *Tle4*, *Dnmt1*, *Cdca5*, and *Brca1* were enriched with the molecular function of chromatin binding. This was consistent with the idea that stem cells that are actively maintaining self-renewal require higher mitotic activity and a greater number of cell divisions. The 37 core genes may play important roles in maintaining a more open chromatin conformation during self-renewal.

**TABLE 1:** The 37 core highly expressed genes.

**Gene Symbol**	**ESC**		**PGC**		**SSC**		**NSC**		**MSC**		**HSC**	
	**logFC**	**adj.P.Val**	**logFC**	**adj.P.Val**	**logFC**	**adj.P.Val**	**logFC**	**adj.P.Val**	**logFC**	**adj.P.Val**	**logFC**	**adj.P.Val**
*Cdca5*	3.36	5.94E−08	3.34	9.93E−08	3.40	6.40E−07	3.20	3.80E−10	1.87	2.95E−06	2.39	1.96E−03
*Trim37*	1.57	2.33E−05	3.58	2.81E−07	3.25	2.31E−07	3.63	6.11E−10	1.82	1.02E−05	2.75	4.13E−05
*Mllt4*	2.39	1.58E−07	1.78	2.31E−06	2.49	1.30E−07	2.46	1.18E−08	1.86	1.44E−06	2.73	1.70E−05
*Chek1*	2.43	6.19E−08	2.72	1.08E−07	2.29	8.29E−06	2.45	1.22E−09	1.75	5.19E−07	2.05	5.84E−04
*Brca1*	3.69	1.82E−08	3.70	5.47E−08	3.42	1.33E−07	2.47	6.81E−10	2.08	6.92E−07	1.70	1.93E−03
*Tle4*	4.93	3.85E−08	5.02	3.54E−08	3.68	1.15E−07	3.48	6.98E−10	1.62	1.53E−05	5.39	2.26E−06
*Rffl*	2.59	3.58E−08	5.14	6.34E−09	3.76	1.01E−08	2.83	2.93E−10	1.85	3.15E−07	5.41	2.52E−06
*Tab3*	1.63	6.47E−07	3.40	3.79E−08	3.23	2.49E−08	2.77	1.94E−10	1.74	6.81E−07	2.86	9.80E−06
*Psmc3ip*	2.45	4.93E−07	4.03	1.08E−07	2.37	7.38E−05	3.11	7.67E−10	1.96	5.42E−06	3.57	7.79E−04
*Cmss1*	2.24	1.85E−07	2.62	1.73E−07	1.75	1.88E−06	2.59	8.82E−10	1.83	3.75E−06	2.30	2.94E−05
*Plekha8*	2.51	1.20E−07	3.30	1.11E−07	3.57	2.08E−08	2.18	2.48E−08	1.66	1.44E−06	1.61	3.86E−04
*Dscc1*	2.89	8.46E−08	3.36	1.18E−07	3.17	5.23E−06	3.52	1.46E−10	2.74	5.41E−07	2.29	1.31E−04
*Dock9*	2.14	1.19E−06	2.82	3.71E−07	4.05	3.95E−08	1.95	4.34E−08	2.10	1.46E−06	3.22	4.17E−05
*Gsta4*	6.86	4.30E−08	4.25	6.85E−07	5.38	8.06E−08	3.27	2.24E−08	3.45	2.07E−06	3.84	1.10E−04
*Incenp*	2.09	1.52E−07	3.17	4.06E−08	2.61	1.84E−06	2.45	1.62E−09	1.81	7.77E−07	2.77	7.76E−04
*Dnmt1*	2.07	1.65E−07	3.83	2.01E−08	2.91	5.57E−08	2.48	1.89E−08	1.85	3.01E−07	4.22	1.26E−05
*Camk1d*	3.36	4.94E−08	2.80	4.35E−07	3.17	6.50E−08	2.17	9.08E−09	3.38	7.90E−08	4.29	1.46E−05
*Tmem170*	2.50	4.25E−07	2.08	4.63E−05	1.83	1.53E−06	2.04	2.07E−08	1.87	2.31E−06	2.91	4.99E−05
*Rad54b*	3.32	2.75E−08	3.24	2.24E−07	3.63	1.31E−06	2.51	3.49E−08	2.11	3.68E−06	3.56	1.05E−05
*Tmem243*	2.50	6.47E−08	4.39	1.24E−08	2.41	2.10E−07	2.00	4.79E−09	2.28	2.13E−07	2.84	7.62E−03
*Foxred2*	2.44	9.71E−07	1.65	1.69E−05	1.99	2.23E−06	2.57	1.52E−08	2.07	1.38E−06	5.34	9.28E−05
*Exoc6*	3.24	4.59E−07	3.56	4.60E−07	2.76	1.34E−06	3.23	2.88E−09	1.93	9.27E−06	6.11	4.21E−06
*Mis18bp1*	2.97	3.58E−08	3.16	7.98E−07	2.82	1.75E−06	2.63	3.13E−09	1.75	1.93E−06	1.60	1.16E−03
*Nav2*	2.85	5.40E−07	2.29	1.87E−05	1.72	3.05E−05	2.57	3.55E−08	1.70	4.39E−05	4.40	4.72E−06
*Rad51ap1*	2.24	3.02E−07	1.84	1.16E−06	1.62	3.62E−05	1.86	5.61E−09	2.07	3.74E−06	2.16	8.17E−05
*Ddb2*	1.76	1.92E−06	3.90	4.36E−08	3.92	5.14E−08	3.10	2.36E−10	1.69	1.49E−06	4.79	7.74E−06
*Cep70*	2.55	3.68E−07	3.78	1.06E−07	3.27	5.16E−07	3.97	1.80E−10	1.88	4.35E−06	4.46	2.83E−06
*Taf7*	4.75	5.74E−09	4.84	2.08E−08	5.48	5.97E−09	1.63	5.81E−08	1.93	3.69E−07	3.74	7.01E−05
*Kbtbd8*	5.09	6.20E−09	5.96	1.11E−08	4.85	3.18E−08	3.91	5.36E−11	2.11	4.64E−06	2.57	3.87E−03
*4930422N03Rik*	2.17	1.96E−06	6.38	9.40E−09	5.34	8.81E−09	2.84	1.18E−09	1.66	3.63E−05	4.01	1.31E−05
*Depdc1b*	2.41	2.73E−05	1.61	3.61E−04	1.98	5.53E−05	3.01	8.87E−08	1.68	2.11E−04	1.72	5.66E−03
*C2cd5*	3.73	5.89E−09	5.03	6.04E−09	2.29	3.75E−07	3.42	4.39E−11	1.86	2.68E−07	3.50	5.27E−05
*Ctnnal1*	6.01	1.78E−08	2.97	8.81E−07	1.81	1.06E−03	3.44	1.80E−09	3.56	7.03E−07	4.29	1.05E−05
*Rps6ka6*	6.29	9.41E−08	4.76	4.13E−07	4.78	3.13E−07	6.22	1.83E−10	3.11	8.37E−06	2.50	3.28E−03
*Rab6b*	2.17	1.14E−05	4.00	4.56E−08	2.53	9.25E−08	5.65	5.49E−12	4.03	8.71E−09	1.57	4.90E−04
*Cdkl5*	1.99	2.48E−05	2.43	7.99E−06	2.78	4.02E−07	3.77	1.42E−10	2.67	7.33E−07	2.10	2.44E−03
*Rps6ka5*	1.55	2.44E−05	2.31	2.37E−07	2.53	8.16E−08	2.59	1.34E−09	2.64	4.85E−07	4.80	1.64E−06

### WGCNA Identified Stem Cell-Specific Co-expression Gene Modules

**FIGURE 2:**
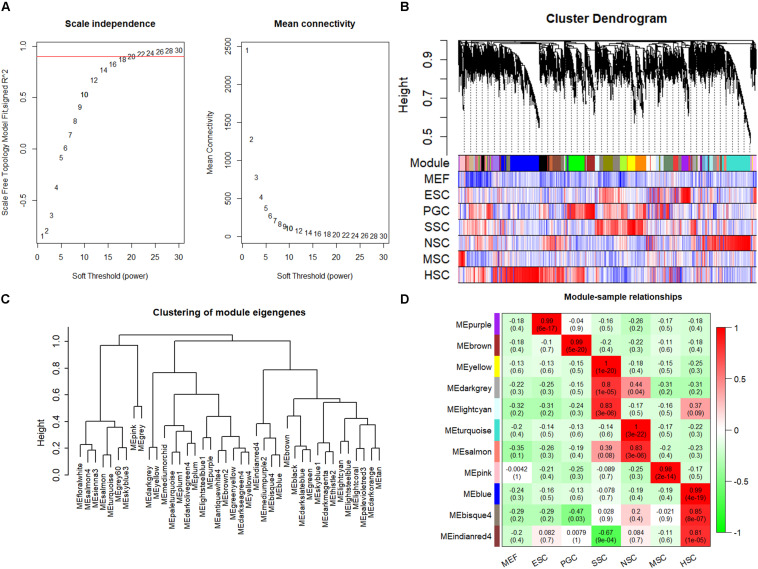
WGCNA identified stem cell type-specific highly expressed genes. **(A)** Selection of soft thresholding powers for WGCNA. The red line corresponds to 0.9. As the lowest power that satisfies the approximate scale-free topology criterion, the number 20 was interpreted as a soft-threshold of the correlation matrix. **(B)** Heat map of the gene dendrogram that was assigned to the six different subtypes of stem cells. The colored row indicates the expression values of genes in the dendrogram. **(C)** The identified module eigengenes were classified into three groups by hierarchical clustering. **(D)** Heat map of the six different subtypes of stem cell-specific modules. Modules with a correlation of >0.80 (*p* < 0.01) were designated as stem cell-specific modules. Each row and column corresponded to the identified module and stem cell type. The correlations and *p*-values of the corresponding modules were labeled.

The respective stem cell-specific genes formed an expression matrix that included 6690 genes. WGCNA analysis was performed to isolate stem cell correlated co-expression genes modules based on the formed expression matrix. The soft threshold power for network construction was selected as 20 (Figure 2A). The eigengenes of modules correlated above 0.85 were merged, and a heat map of the gene dendrogram was assigned to the six different subtypes of stem cells (Figure 2B). From the heatmap, we can easily obtain information of cluster genes which were highly expressed with stem cell types. Gene module eigengenes were classified into three groups by hierarchical clustering (Figure 2C). Modules correlated to stem cell types that possessed correlations of above 0.80 were selected for further study. We found 11 modules that were correlated to the six stem cell types based on the correlation between modules and respective stem cell types (Figure 2D). Specifically, purple was correlated to ESCs, brown was correlated to PGCs, yellow, light cyan, and dark gray modules were correlated to SSCs, turquoise and salmon modules were correlated to NSCs, the pink module was correlated to MSCs, and blue, bisque4, and indianred4 were correlated to HSCs. WGCNA identified stem cell special co-expression networks which make it possible to detail the mechanisms of stem cell maintenance.

### Function Annotation of Stem Cells Types Respective Modules

**FIGURE 3:**
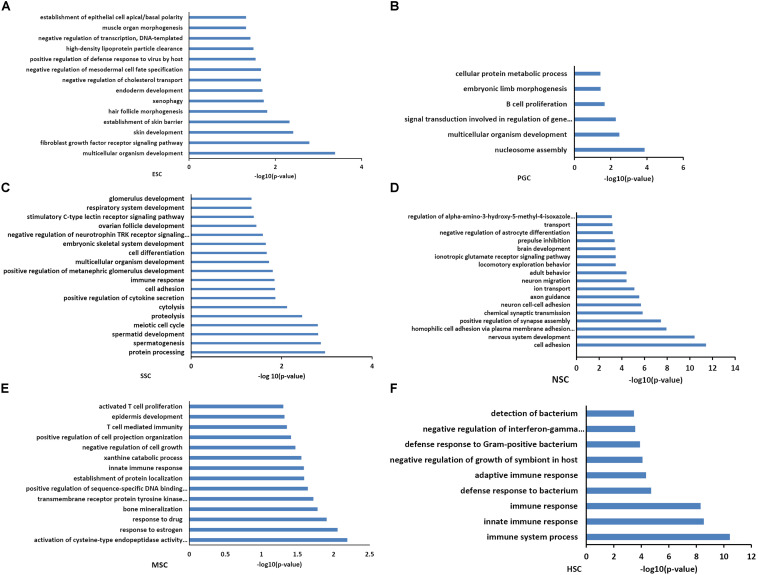
Functional annotation of the six stem cell types correlated modules. GO analysis of genes in six stem cell types correlated modules were annotated by DAVID 7.8. ESC enriched GO terms included multicellular organism development, endoderm development, and the establishment of various organs and cells **(A)**. GO analysis of PGCs correlated modules genes were involved in multicellular organism development and embryonic limb morphogenesis **(B)**. SSC correlated module genes were involved in cell differentiation, spermatogenesis-related processes, and multicellular organism development **(C)**. NSC correlated module genes were involved in neural system development **(D)**. MSC correlated module genes were involved in T cell development, T cell mediated immunity, immune responses, and bone mineralization **(E)**. HSC correlated module genes were involved in the immune response **(F)**.

Functional annotations of the respective types of stem cells correlated modules were performed by DAVID system. The enriched GO_BP terms were consistent with their respective stem cell characteristic properties. The purple module, which is related to ESCs, was involved in multicellular organism development, endoderm development, and the establishment of various organs and cells (Figure 3A). This was consistent with the multi-lineage differentiation potential of ESCs. Brown and yellow module genes were also involved in multicellular organism development, indicating that PGCs and SSCs could obtain ESC-like features and differentiate into progeny cells of three different germ layers (Figures 3B,C). SSCs correlated yellow module genes were involved in spermatogenesis-related processes, and SSCs possessed characteristic properties of adult germline stem cells. NSC correlated turquoise module genes were involved in neural system development, which was consistent with neural stem cell characteristic properties (Figure 3D). MSC correlated pink module genes were involved in T cell development, T cell mediated immunity, immune responses, and bone mineralization which exactly demonstrated the bone marrow derivation of MSCs (Figure 3E). HSC correlated blue module genes were involved in the immune response, and this was consistent with the characteristic properties of HSC (Figure 3F). Functional annotations of the respective types of stem cells correlated modules consistent with the stem cell properties means that the association of co-expression genes modules with respective stem cells is feasible.

### Network Analysis of the Stem Cells Respective Modules

Given that the genes possessed high connectivity in stem cell correlated modules, these genes may play similar roles in maintenance of stem cell properties. A subsequent analysis was performed on these high-connectivity genes. The connectivity threshold of the edges between two genes was set at 0.4 for turquoise (253 genes), 0.3 for blue (363 genes), 0.2 for brown (148 genes), yellow (130 genes) and purple (127 genes), and at 0.05 for pink (125 genes). The genes within the respective stem cell-specific modules are listed in Supplementary Table S3. The topological analysis of the co-expression network provided information for the maintenance of stem cell properties maintaining. According to topologic principles, hub genes with high degrees of connectivity played important roles in continuous network. We displayed the connections of those co-expression genes by Cytoscape software.

**FIGURE 4:**
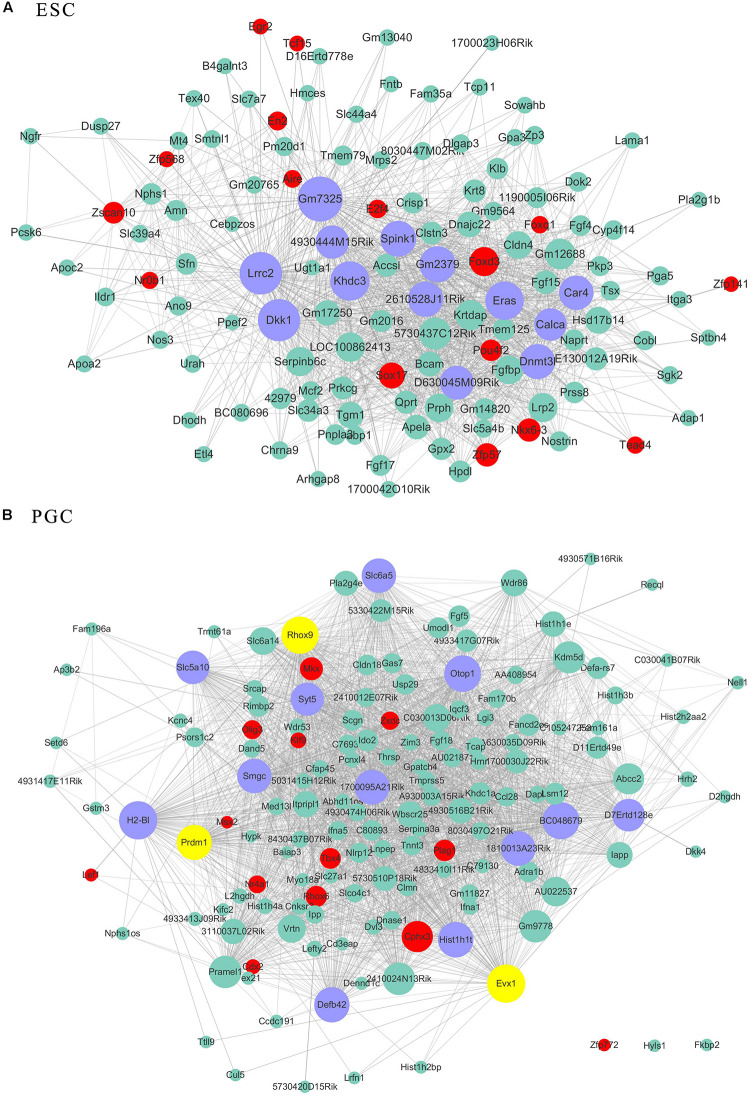
The related co-expression networks of ESC and PGC. The co-expression networks of the ESC-correlated purple module **(A)**, PGC-correlated brown module **(B)**. The hub nodes are highlighted in blue within the co-expression networks. Transcription factors within the respective network are highlighted in red. Transcription factors that were included in the top 10% of nodes are highlighted in yellow within the respective networks. The size of the nodes corresponds to their degrees.

**FIGURE 5:**
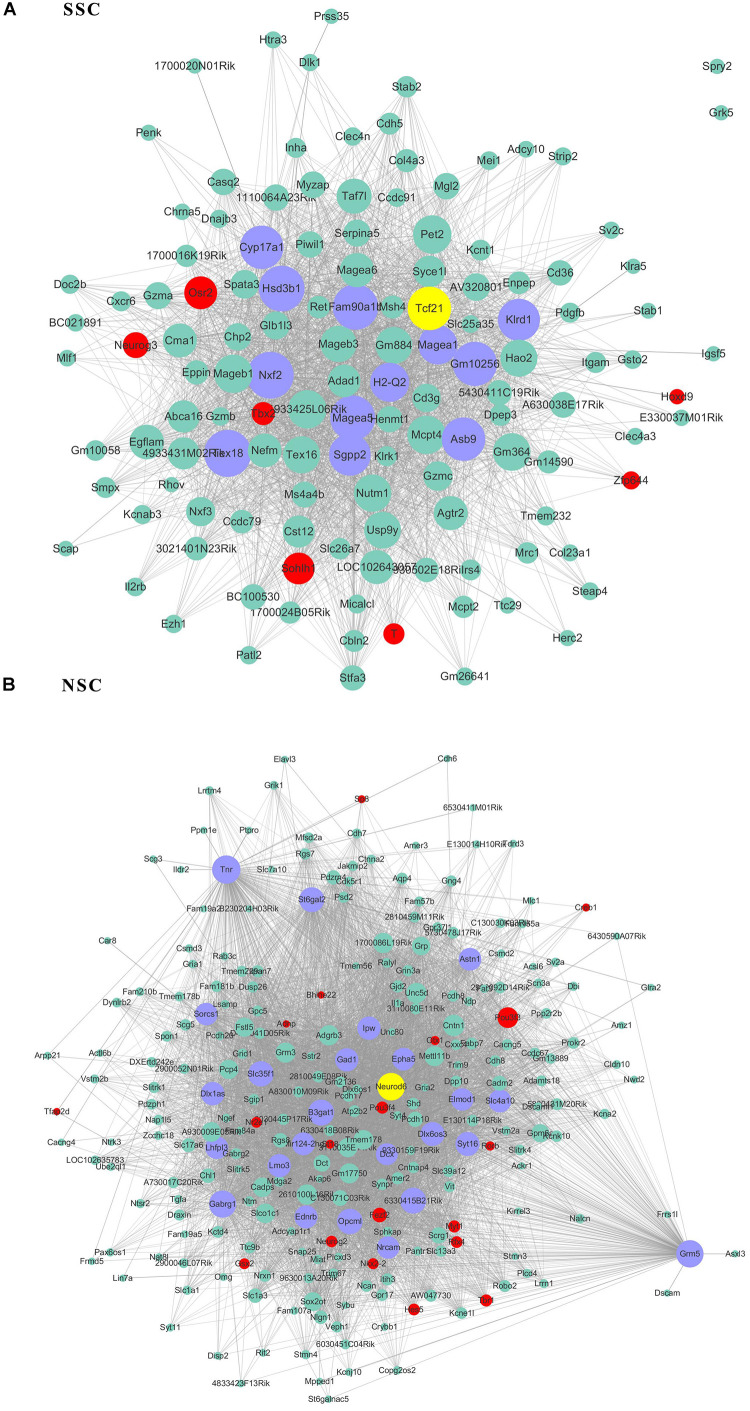
The related co-expression networks of SSC and NSC. SSC-correlated yellow module **(A)**, NSC-correlated turquoise module **(B)**. The hub nodes are highlighted in blue within the co-expression networks. Transcription factors within the respective network are highlighted in red. Transcription factors that were included in the top 10% of nodes are highlighted in yellow within the respective networks. The size of the nodes corresponds to their degrees.

**FIGURE 6:**
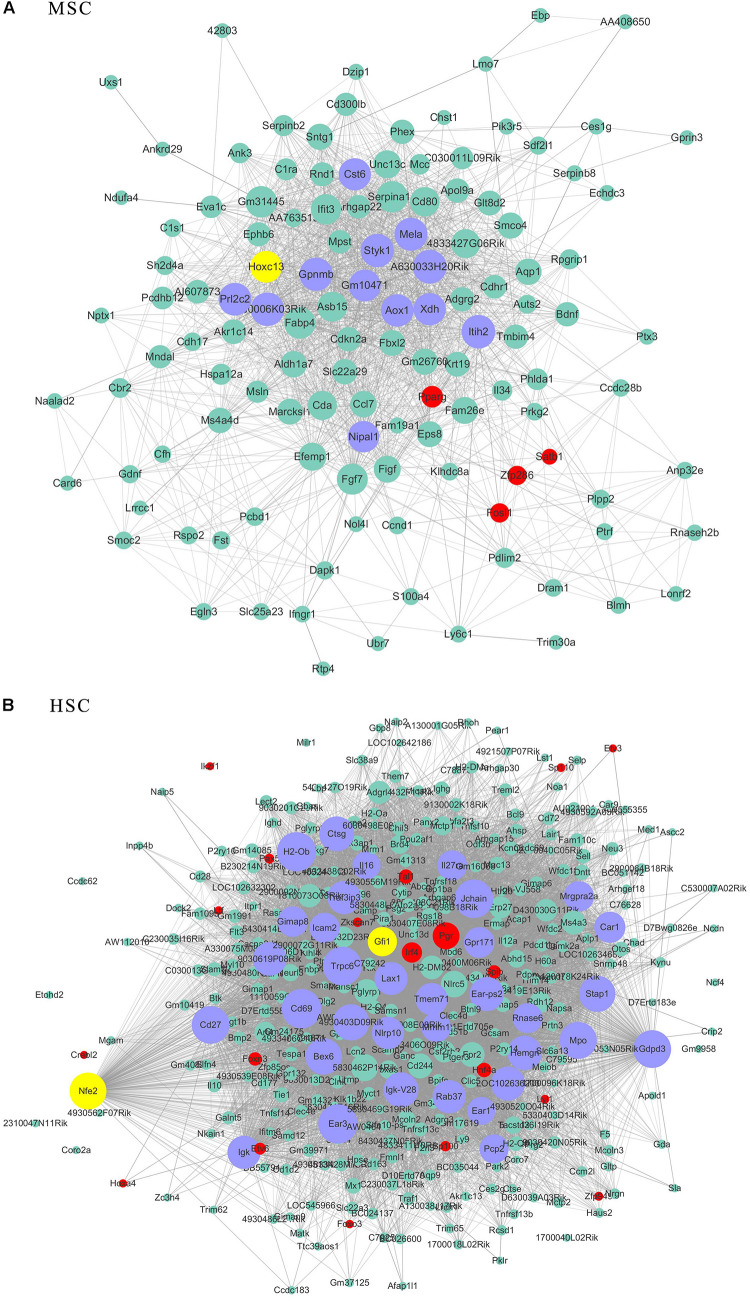
The related co-expression networks of SSC and NSC. MSC-correlated pink module **(A)** and HSC-correlated blue module **(B)**. The hub nodes are highlighted in blue within the co-expression networks. Transcription factors within the respective network are highlighted in red. Transcription factors that were included in the top 10% of nodes are highlighted in yellow within the respective networks. The size of the nodes corresponds to their degrees.

As transcriptional factors play important roles in maintaining cell types, we focused our studies on the genes within the connectivity network that were involved in transcriptional regulation. The results from this analysis are presented in Figures 4–6. Transcription related genes are highlighted in red. Hub genes that were within the top 10% of the high connectivity network are highlighted in blue, and these are presented in the respective module networks generated by topological analysis. The transcription factors *Foxd3* and *Zscan10* aligned with the hub genes within the purple module were required to maintain mouse ESCs (Hanna et al., 2002; Yamane et al., 2015; Figure 4A). The *Zscan10* was also found to be a key component of the ESC core transcriptional regulatory network formed by *Pou5f1* and *Sox2* (Yu et al., 2009). Transcriptional repressor *Prdm1* (Yamashiro et al., 2016), transcription factors *Rhox9*, and *Evx1* were aligned with the hub genes of the brown module means those transcription factors mayplay an essential role in PGC specification (Figure 4B). *Tcf21* was aligned with the hub genes of the yellow module (Figure 5A). The expression of *Tcf21* was higher in SSC that may be consistent with regulation of sex determining factor SRY. The transcription factor *Neurod6* was aligned with the hub genes within the turquoise module (Figure 5B). *Neurod6* is known to be involved in retina cell fate determination, differentiation, morphological development, and circuit formation (Cherry et al., 2011). *Hoxc13* was aligned with the hub genes of pink module (Figure 6A). The transcription factors *Nfe2* and *Gfi1* were aligned with the hub genes of the blue module (Figure 6B), indicating that *Nfe2* and *Gfi1* are important for the maintenance of HSCs. *Nfe2* was found to be required for adult thrombocyte formation and function in zebrafish (Rost et al., 2018). Transiently expressed *Gfi1* could convert adult murine endothelial cells to hematopoietic stem cells (Barcia Duran et al., 2018). After topological analysis of the co-expression network, we will easily obtain useful clues for stem cell maintenance. Since *Foxd3* and *Zscan10* have been found playing important roles in maintaining mouse ESCs. There are reasons to speculate that *Zfp57*, *Pou4f2*, *Sox17*, and *Nkx6-3* which are the subsequent predicted transcription factors also vital to maintenance of mouse ESCs. In the same way, other predicted transcription factors in stem cell special co-expressed network would be critical for maintenance stem cell pool.

**FIGURE 7:**
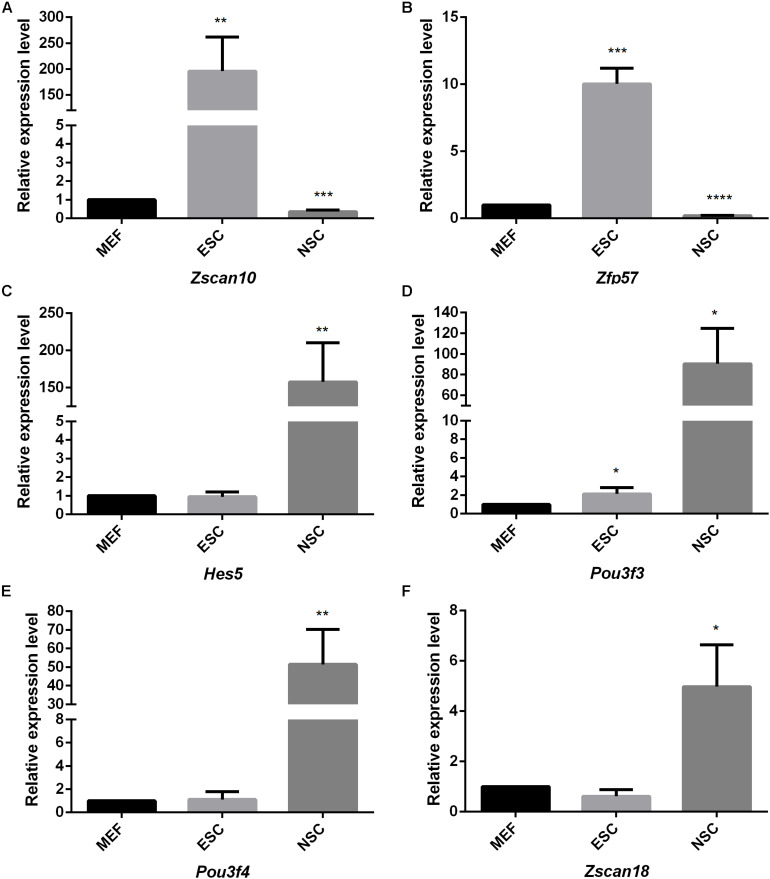
The quantitative analysis of identified stem cell related genes based on qPCR **(A–F)**. The expression levels of identified stem cell related genes were detected in ESC and NSC. All data were normalized to the β-actin and are displayed as fold changes compared to MEF. Error bars are the standard deviation (SD) of at least three repeats. The significance of differences in multiple comparisons were determined by student’s *t*-test. “*” means *p* < 0.05, “**” means *p* < 0.01, “***” means *p* < 0.001, and “****” means *p* < 0.0001.

Considering the thousands of different expressed genes obtained from difference analysis, stem cell correlated co-expression network provide clues to detail regulatory networks of key transcription factors. We intend to detect the expression of the identified stem cell related genes by qPCR detection. Pluripotent transcription factor *Pou5f1* and NSCs special marker *Nestin* were used to confirm the respective identities of ESCs and NSCs. Both of them were lowly expressed in MEF. The identified ESC correlated transcription factors *Zfp57* and *Zscan10* were highly expressed in ESC compared to MEF. Both of *Zfp57* and *Zscan10* lowly expressed in NSC means that *Zfp57* and *Zscan10* may not be required in neurogenesis during embryo development (Figures 7A,B). We also detected *Hes5*, the Notch signaling effector which acts as a key regulator of maintenance of NSC was highly expressed in NSC (Figure 7C). NSC correlated transcription factors *Pou3f3*, *Pou3f4*, and *Zscan18* were also more highly expressed in NSC compared to MEF (Figures 7D–F). The expression levels of several identified stem cell related transcription factors were consistent with our prediction.

**FIGURE 8:**
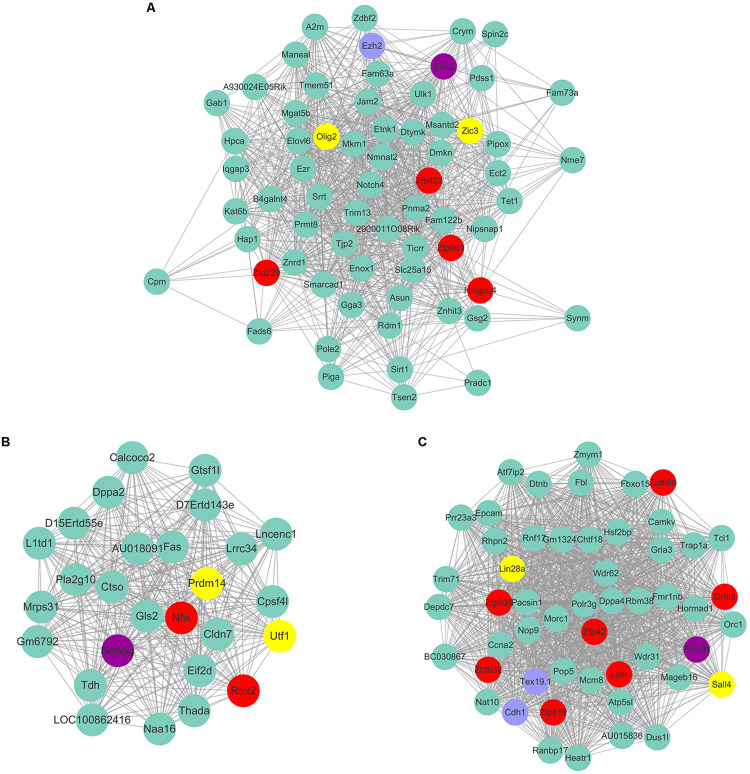
The predicted co-expressed network of Yamanaka factors. The co-expression networks of Yamanaka factors Sox2 **(A)**, Nanog **(B)**, and Pou5f1 **(C)**. Yamanaka factors Sox2, Nanog and Pou5f1 are labeled in purple. Several well-known stemness transcription factors are marked in yellow and non-transcription factors were labeled as blue. The predicted transcription factors within the respective network of respective Yamanaka factors are highlighted in red. For clarity, the top 20% of the nodes of Nanog and Pou5f1 co-expressed genes were displayed according to node degree.

As Yamanaka factors are famous for promoting MEF transdifferentiated into iPS, we then attempted to determine whether or not the transcription factors listed in ESC are most correlated with the purple module. Unfortunately, the well-known transcription factors are not in the list of ESC’s most correlated module genes. Although *Pou5f1 (Oct4)*, *Sox2*, *Nanog* are not listed in ESC most relevant modules, they are part of the respective modules that are relatively highly correlated to ESC. For example, Sox2 belongs to the module Plum1 with a correlation of 0.78 (*p* value = 6.36E-05). Nanog belongs to the module lightsteelblue1 with a correlation of 0.62 (p value = 0.0026). *Pou5f1* belongs to the module Yellow4 with a correlation of 0.57 (p value = 0.0064). Unfortunately, the other Yamanaka factors *Klf4* and *Myc* are in the modules brown2 and Lightcoral, with no significant correlation (brown2, cor = −0.1, p value = 0.7; Lightcoral, cor = 0.26, p value = 0.2). It is also worth mentioning that *Klf4* was listed in the PGC-correlated brown module with a correlation of 0.78 (p value = 3E-05). *Klf4* was found to be expressed at a high level in human PGCs, and transfection with both *Sox2* and *Pou5f1* resulted to inducing PGCs into iPS at a relatively high efficiency (Wei et al., 2009). The Yamanaka factors listed in different respective modules may reflect the different regulatory networks among those transcription factors. Several of those neighbor nodes were famous transcription factors related to the maintenance and transdifferentiation of ESC, such as *Sox2* interacted with *Dppa5a*, *Zic3*, and *Peg10* (Figure 8A). *Zic3* enhances the generation of mouse iPS depend on interact with *Pou5f1*, *Sox2* and *Klf4* (Declercq et al., 2013). Histone methyltransferase *Ezh2* that required for stable ESC self-renewal and differentiation also lists in the *Sox2* mediated network. *Nanog* interacted with *Prdm14, Nifx*, and *Rex2* (Figure 8B). *Prdm14* were found playing important roles in induced pluripotent stem sell reprogramming (Seki, 2018). *Pou5f1* interacted with *Sall4*, *Lin28a* (Figure 8C). *Pou5f1* interacted with *Sall4* formed a transcription regulation feedback loop governing the “stemness” of ES cells (Yang et al., 2010). The predicted transcription factors within the respective network of respective Yamanaka factors which highlighted in red may be playing important roles in maintenance and transdifferentiation of ESC.

### Validation of Stem Cell-Specific Modules Based on STRING Database

To validate the co-expression connections of stem cells correlated modules genes, protein-protein interaction networks were detected by STRING v11 (Szklarczyk et al., 2019). The six types of stem cell correlated genes and the 37 core genes were imported into the STRING database to generate a continued protein-protein interaction network that was bridged by 25 other genes. The continued protein-protein interaction network contains 823 nodes and 3113 edges (Supplementary Figure S3).

**FIGURE 9:**
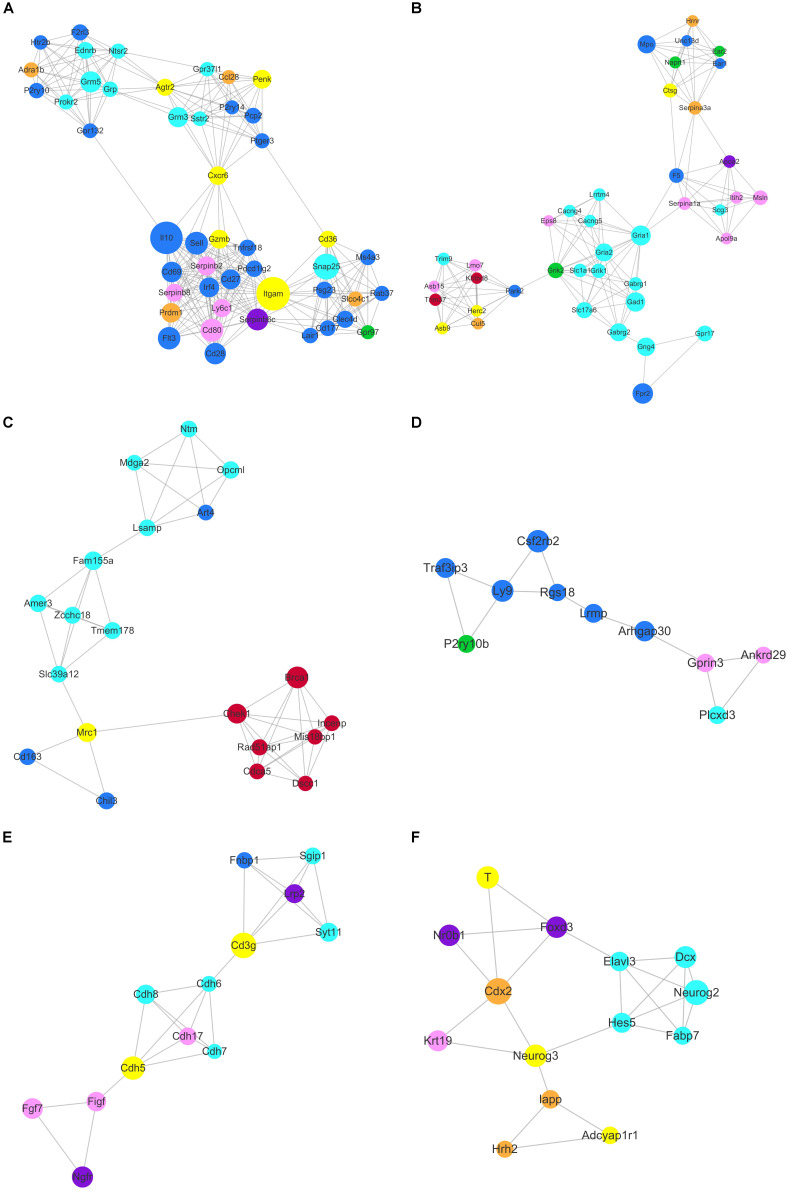
Identification of six densely connected regions within the constructed stem cell correlated protein-protein interaction network based on the STRING database. Six densely connected regions were identified within the continued protein-protein interaction network by topology analysis **(A–F)**. The nodes are marked with the colors purple (ESC), brown (PGC), yellow (SSC), turquoise (NSC), pink (MSC), and blue (HSC) corresponding to the respective stem cell correlated modules.

Based on topology, we identified six densely connected regions within the continued protein-protein interaction network (Figure 9). Densely connected regions provide useful information with regard to the ability of stem cells to maintain their respective characteristic properties. Validated protein-protein interactions confirmed that co-expressed genes formed independent interaction modules. The identification of the interactions among different stem cell correlated genes would provide useful insights into the molecular mechanisms of transdifferentiation. The SSC correlated genes *Itgam*, *Cxcr6*, and *Agtr2* bridged four densely connected regions that consisted primarily of HSC, NSC, and MSC correlated genes (Figure 9A). As predicted transcription factors *Dnmt1* and *Prdm1* have been found essential for SSCs maintenance, those cell surface genes may participate in some signal pathway activating the expression of transcription factors. Those cell surface genes are essential to maintain stem cell niche.

The core genes *Chek1*, *Brca1*, *Cdca5*, *Rad51ap1*, *Mis18bp1*, *Incenp*, and *Dscc1* formed densely connected modules that were linked to the NSC correlated gene network via Mrc1 in Figure 9C. The majority of the seven genes were annotated with cell cycle and chromatin. *Zcchc18* acts as transcriptional factor within the NSC related gene interaction network. Wnt signaling plays an important role in stem cell maintenance by targeting various cadherin molecules such as *Cdh6*, *Cdh7* and *Cdh8* in NSCs, *Cdh5* in SSCs, and *Cdh17* in MSCs (Figure 9E). As indicated in Figure 9F, the neural related genes *Hes5*, *Neurog2*, *Dcx*, *Fabp7*, and *Elavl3* form a densely connected region that may be critical for NSC maintenance. The connected network consisted of ESC, PGC, and SSC related genes. The dynamics expression of *Hes5* was correlated with cell fate determination during embryonic development (Munoz-Esquivel et al., 2019). *Hes5*, *Neurog3*, *Cdx2*, *Foxd3*, *Neurog2*, and *T* were the transcription factors that mediated the bulk of the downstream gene expression. Manipulating the expression of those transcription factors may provide useful information for stem cell transdifferentiation *in vivo*.

### CCCTC-Binding Factor (CTCF) Motif Analysis Provide Clues for the Global Organization of Chromatic Architecture of Stem Cells

**FIGURE 10:**
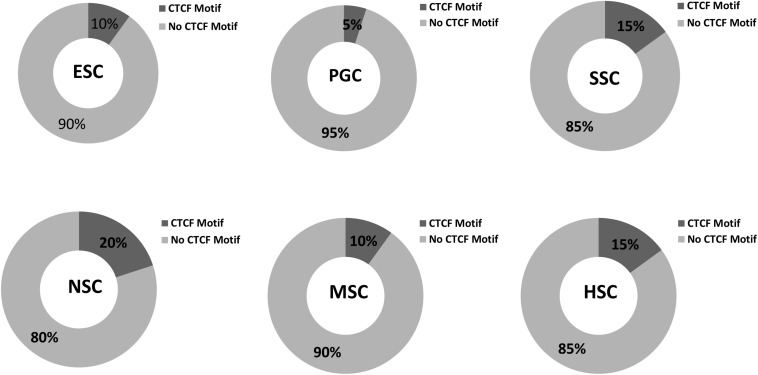
CTCF binding sites in respective stem cell hub genes. The occupancy rates of the CTCF binding motif among the 20 hub genes within the respective stem cell specific modules. Dark gray shading indicates the percentages of CTCF binding motif among the 20 hub genes in the six stem cell types. Light gray corresponded to non- CTCF binding motifs.

Various cell types and physiological states typically display different chromatin structures. Given the highly specific transcriptional activity of stem cells, chromatin structures within these stem cells may display different features. The GO analysis of the 37 common highly expressed core genes enriched in DNA recombination and chromatin organization indicated that CTCF regulates the structure of chromatin and defines the boundaries between active and heterochromatic DNA. This finding prompted us to evaluate the CTCF binding sites within those hub genes. As shown in Figure 10, the results indicated that 5–20% of the hub genes possessed a binding motif for CTCF, and this is relatively lower than the value of 33% that is expected for genome-wide CTCF binding sites (Holwerda and de Laat, 2013). Random samplings of 20 genes from the expression matrix displayed random occupancy rates of the genome-wide CTCF binding sites (Supplementary Figure S4). This indicates that stem cells require chromosome opening activity to successfully maintain stem cell-specific stemness properties. Highly expressed genes are typically located far from the boundaries between active and heterochromatic DNA. It is also possible that the few hub genes that possess CTCF binding sites may be more rigorously regulated during stem cells differentiation. It is established that PGCs possess a special germ-line differentiation potential and exhibits the lowest number of CTCF binding motifs within their correlated hub genes. NSC correlated hub genes possess the highest number of CTCF binding motifs, suggesting that neural cell differentiation requires chromatin remodeling. This is consistent with the findings of a previous study that the activation of neuronal specific genes requires chromatin modification (Su et al., 2017).

### ESC and NSC Shown More Conservative Than Other Types of Stem Cells Between Human and Mouse

As the 37 core genes co-expressed in multiple types of stem cell in mouse, we also detected the expression profiles of different types of stem cells in human. Further analysis found that only partial of mouse stem cell up-regulated genes were detected in human respective stem cells due to differences between species. There are 35 human homologous genes according to mouse core 37 genes. Whether or not of those core genes were up-regulated in the respective human stem cells were shown in Supplementary Table S4. Human ESC up-regulated genes contain 14 of 37 core genes and human NSC and SSC respective contain 8 and 12. CDCA5, DEPDC1B, RAD51AP1 and RAD54B were all up-regulated in human ESC, NSC, and SSC. To our surprise, none of the 37 core genes was up-regulated in human MSC. The percentages of common up-regulated genes both in mouse and human stem cells are different among the 5 types of human stem cells (Supplementary Figure S5). ESC and NSC shared more conservative between human and mouse. SSC and HSC are moderate conservative. Human MSC displays distinct signatures, only 27 genes were both up-regulated in mouse and human. That maybe explain none of the 37 core genes was up-regulated in human MSC. Higher common up-regulated genes both in mouse and human ESC and NSC may be explained by the conservative mechanism of mammalian embryo and neural system development. Most famous stem cell marker genes were conservative in both mouse and human. For example, homologous genes of famous ESC special transcription factor *Pou5f1*, *Lin28a*, *Nanog*, *Sox2* were also up-regulated in human ESC. NSC special genes *Sox2*, *Olig1* and *Pou3f3* were also up-regulated in human NSC. The same as SSC special genes were also up-regulated in human SSC, such as *Epcam*, *Ddx4*, *Tex14*, *Cdh1*, *PIWIL2* and so on. The relative expression foldchanges of those homologous genes were annotated in Supplementary Table S5.

Next, we also validated whether or not of those stem cell special genes which identified by WGCNA were also up-regulated in respective human stem cells. Almost half of mouse NSC special genes were also up-regulated in human NSC. As mouse NSC special turquoise module enrich 253 genes, there are 207 human homologous genes contains 97 genes that up-regulated in human NSC. The percentages of homologous genes in mouse NSC special modules was higher than common up-regulated genes shared by mouse and human (46.9% vs. 27.9%). Some identified transcription factors were also detected in corresponding human stem cells. For example, qPCR validated NSC correlated transcription factors *Pou3f3* was also up-regulated in human NSC. Other NSC special transcription factor *Hes5*, *Rfx4* and *Nkx2-2* were also up-regulated in human NSC. qPCR validated ESC correlated transcription factors *Zscan10* and ESC self-renewal related transcription factor *Foxd3* were also up-regulated in human NSC. Further exploring function of identified conservative homologous genes may provide clues for detailing differences between species development. The percentages of homologous genes of mouse NSC special modules was higher than common up-regulated gene means that results of WGCNA data mining are credible. Those identified mouse stem cell special genes which also up-regulated in human stem cells were highlighted with yellow color in Supplementary Table S3.

## Discussion

*In vivo* cell fate reprogramming has emerged as a new method for understanding cell plasticity and as potential treatment for tissue regeneration. Understanding the underlying mechanism controlling cell fate reprogramming would promote the development of more accurate and efficient strategies for tissue regeneration and repair. Although there are many studies examining stem cell renewal, the specific mechanism underlying the maintenance of stemness in several adult stem types remains unknown. Here, we aimed to identify the key genes associated with ESCs, PGCs, SSCs, NSCs, MSCs, and HSCs. A total of 37 core genes were highly expressed in all of the above stem cells. These genes were identified, and stem cell correlated gene co-expression networks were obtained. The validation of the co-expression genes among different stem cells allowed us to construct a continued protein-protein interaction network that included 823 nodes and 3113 edges. Based on topology, six densely connected regions within this continued protein interaction network were found. Densely connected regions provide useful information with regard to the characteristic properties of stem cell maintenance. The interactions among different stem cell related genes provided useful information regarding the process of transdifferentiation. CTCF binding motif analysis revealed that the hub genes of respective stem cell modules occupy less CTCF. Clarifying the mechanisms underlying adult stem cell self-renewal will aid in our understanding of stem cell pool maintenance and will promote the development of more accurate and efficient strategies for tissue regeneration and repair.

The 37 core genes may play important roles in maintaining an open chromatin conformation during self-renewal. Those core genes may determine the difference between stem cells and terminally differentiated cells. *Trim37* monoubiquitinates histone H2A, and this process is associated with transcriptional repression (Bhatnagar et al., 2014). *Trim37* may repress various genes during stem cell self-renewal. The observation that the ubiquitin ligases BRCA1 and CHEK1 are both highly expressed within stem cells indicates that DNA repair mechanisms are highly active in the maintenance of stem cell self-renewal. The nucleotide excision repair protein DDB2 is highly expressed in the stem cells, suggesting that the nucleotide excision repair mechanism may also be essential for the maintenance of stem cell genome integrity. *Dnmt1* was also identified as one of the 37 core genes, indicating that DNA demethylation is essential for stem cell maintenance. The general transcription factor *Taf7* has been found to play an essential role in embryonic development (Gegonne et al., 2012). *Taf7* was included in the 37 core genes, which means that *Taf7* is also essential for SSC, NSC, MSC, HSC, and PGC maintenance.

Several of those neighbor nodes in Yamanaka factors belonged to modules were famous transcription factors related to the maintenance and transdifferentiation of ESC, such as *Sox2* interacted with *Dppa5a*, *Zic3*, and *Peg10*. *Nanog* interacted with *Prdm14*, *Nifx* and *Rex2*. *Pou5f1* interacted with *Sall4*, *Lin28a*. The expression levels of several identified stem cell related transcription factors were consistent with our prediction. Based on the bioinformatic prediction and qPCR detection, we think it is worth to reprogram MEF to ESC or NSC by overexpression of *Zfp57* or *Zscan18*.

HSC transplantation is well characterized as the first widely accepted adult stem cell within the hematopoietic system that could give rise to different types of blood cells. In recent years, studies have found that HSC transplantation can not only produce cells within the blood system but can also improve body function. Bone marrow cells migrate into the liver, and they fuse with hepatocytes to produce proliferating cells that are responsible for liver regeneration (Pedone et al., 2017). Transplanted bone marrow cells can also fused with neuronal cells in murine adult brains to protect and regenerate brain tissues (Weimann et al., 2003). Based on their diversity and differentiation potential, MSCs have gained increasing attention for their potential use in tissue repair and regeneration. The validation of the stem cell correlated modules that were generated based on the STRING database provided useful information regarding the characteristic properties of stem cell maintenance. Identifying the interactions among different stem cell correlated genes provided useful insights into the process of transdifferentiation. Although none common gene was shared in the all of the 5 types of human stem cells, ESC and NSC shown more conservative than others between human and mouse. Most of the ESC and NSC shared more conservative between human and mouse. The percentages of homologous genes of mouse NSC special modules was higher than common up-regulated gene means that results of WGCNA data mining are credible. Further detailing the conservative transcription factors between mouse and human may be provide clues for such as *Pou3f3*, *Hes5*, *Rfx4* and *Nkx2-2* for NSC, *Zscan10, Foxd3* for ESC. Manipulating the expression of those identified transcription factors may provide useful information that will allow for a greater understanding of stem cell transdifferentiation.

## Conclusion

Highly efficient and precise reprogramming requires an understanding of the transcriptomes which function within different cell types. In this study, we focus on the underlying molecular mechanisms of self-renewal in several well-known stem cell types. We identified 37 core genes that were highly expressed in all of the stem cell types examined, and stem cell correlated gene co-expression networks were obtained based on WGCNA. The validation of the co-expression genes among different stem cell types revealed a continued protein-protein interaction network that included 823 nodes and 3113 edges. Based on topology, densely connected regions provide useful information with regard to the ability of stem cells to maintain their respective characteristic properties.

CTCF binding motif analysis revealed that the hub genes of respective stem cell modules occupy less CTCF. Human stem cells display a distinct result from mouse stem cell. ESC and NSC shown more conservative than other types of stem cells between human and mouse. Clarifying the mechanisms underlying adult stem cell self-renewal will aid in the understanding of stem cell pool maintenance and will promote more accurate and efficient strategies for tissue regeneration and repair.

## Data Availability Statement

All datasets presented in this study are included in the article/Supplementary Material.

## Ethics Statement

The animal study was reviewed and approved by Animal Ethics Committee of Shandong University of Technology.

## Author Contributions

W-HX and LS: conceptualization and supervision. L-JW, X-XL, and W-HX: methodology. L-JW, X-XL, and JH: software and formal analysis. L-JW, X-XL, JH, and X-HS: investigation. X-HS and W-HX: resources. L-JW and W-HX: data curation. L-JW and X-XL: writing-original draft preparation. W-HX: writing review and editing and project administration. L-JW, LS, and W-HX: funding acquisition. All authors contributed to the article and approved the submitted version.

## Conflict of Interest

The authors declare that the research was conducted in the absence of any commercial or financial relationships that could be construed as a potential conflict of interest.

## Supplementary Material

FIGURE S1 The number of genes only in the respective stem cell in contrast to up-regulated genes.

FIGURE S2 Heatmap of the expression relative value of the 37 core genes in the respective stem cells and MEF.

FIGURE S3 The continued protein-protein interaction network contains 823 nodes and 3113 edges. The nodes were painted as colors such as turquoise, blue, brown, yellow, purple, and pink corresponding to the stem cell-respective modules.

FIGURE S4 Random occupancy rates of the CTCF binding motif. Random sampling was used to evaluate the genome-wide CTCF binding sites.

FIGURE S5 The percentages of common up-regulated genes both in mouse and human stem cells are different among the 5 types of human stem cells. Mouse_homolog means the up-regulated genes of mouse respective stem cells corresponding to human homologous genes. The percentage was calculated by compared gene amount of respective sections to the sum of mouse and human up-regulated genes.

TABLE S1 Primer sequences used in this study.

TABLE S2 The highly expressed gene lists of each types of stem cell.

TABLE S3 The gene lists of stem cells respective special modules. Those identified mouse stem cell special genes which also up-regulated in human stem cells were highlighted with yellow color.

TABLE S4 Whether or not of those core genes were up-regulated in the respective human stem cells.

TABLE S5 The relative expression foldchanges of those annotated homologous genes between mouse and human.
