# Supplementary material for: Integrated Analyses of Mouse Stem Cell Transcriptomes Provide Clues for Stem Cell Maintenance and Transdifferentiation
Source: Front Genet. 2020 Sep 4;11:563798. doi: 10.3389/fgene.2020.563798 (PMC7500244; doi:10.3389/fgene.2020.563798)
Supplement: FIGURE S1 — The number of genes only in the respective stem cell in contrast to up-regulated genes. [file Data_Sheet_1.docx]

Supplementary Material

# Supplementary Data

Supplemental Figure S1: The continued protein-protein interaction network contains 823 nodes and 3113 edges. The nodes were painted as colors such as turquoise, blue, brown, yellow, purple and pink corresponding to the stem cell-respective modules.

Supplementary Figure S2**.** Heatmap of the relative expression of the 37 core genes in the respective stem cells and MEF.

Supplementary Figure S3: A continued protein protein interaction network that was bridged by 25 other genes.

Supplementary Figure S4. Random occupancy rates of the genome-wide CTCF binding sites.

Supplementary Figure S5. The percentages of common up-regulated genes both in mouse and human stem cells are different among the 5 types of human stem cells.

Supplementary Table S1. Primer sequences used in this study.

Supplementary Table S2. The highly expressed gene lists of each types of stem cell.

Supplementary Table S3. The gene lists of stem cells respective special modules. Those identified mouse stem cell special genes which also up-regulated in human stem cells were highlighted with yellow color.

Supplementary Table S4. Whether or not of those core genes were up-regulated in the respective human stem cells.

Supplementary Table S5. The relative expression foldchanges of those annotated homologous genes between mouse and human.

# Supplementary Figures and Tables

**Supplementary Figures**





**Supplementary Figure 1.** The number of genes only in the respective stem cell in contrast to up-regulated genes.


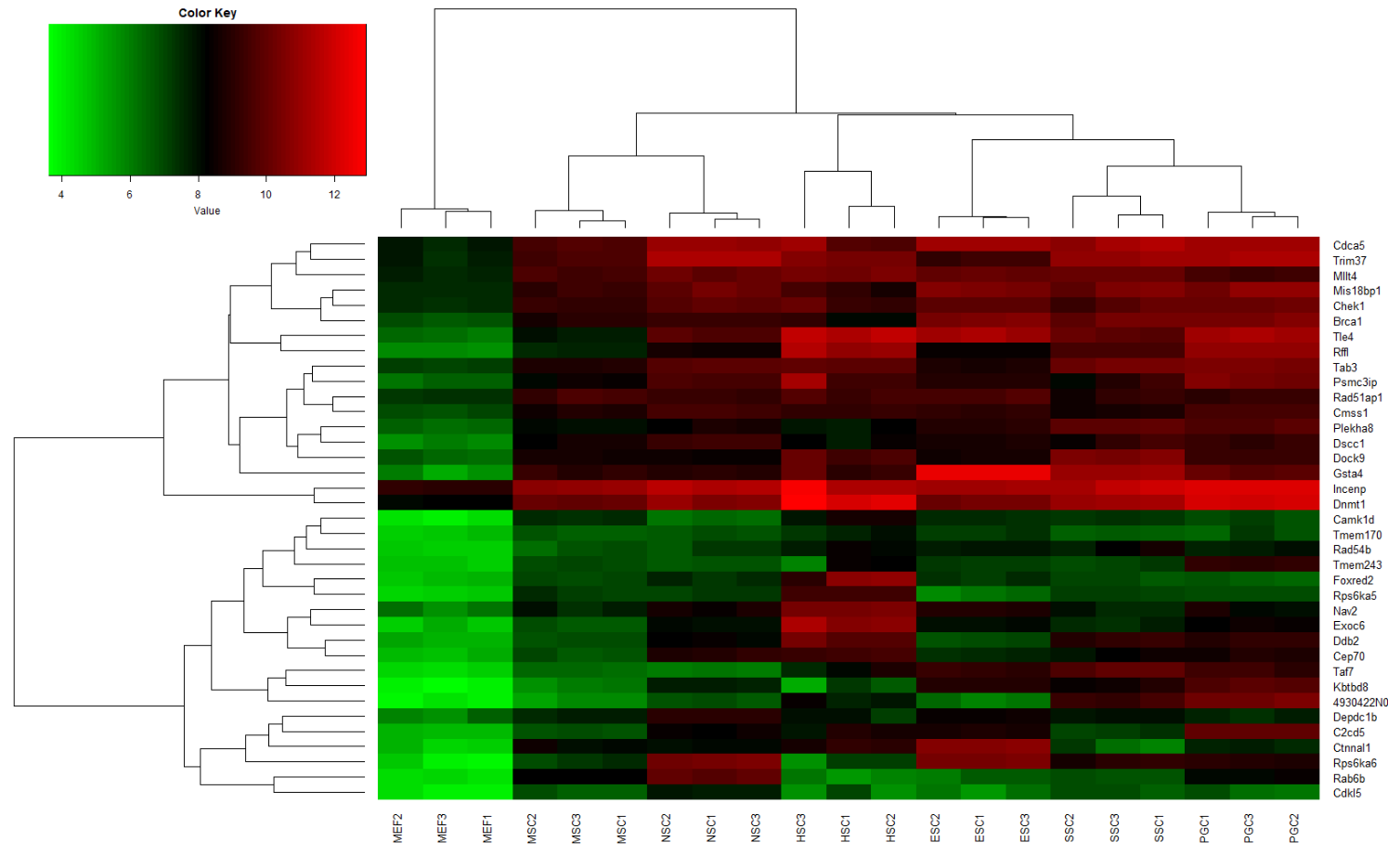


**Supplementary Figure 2.** Heatmap of the relative expression of the 37 core genes in the respective stem cells and MEF.


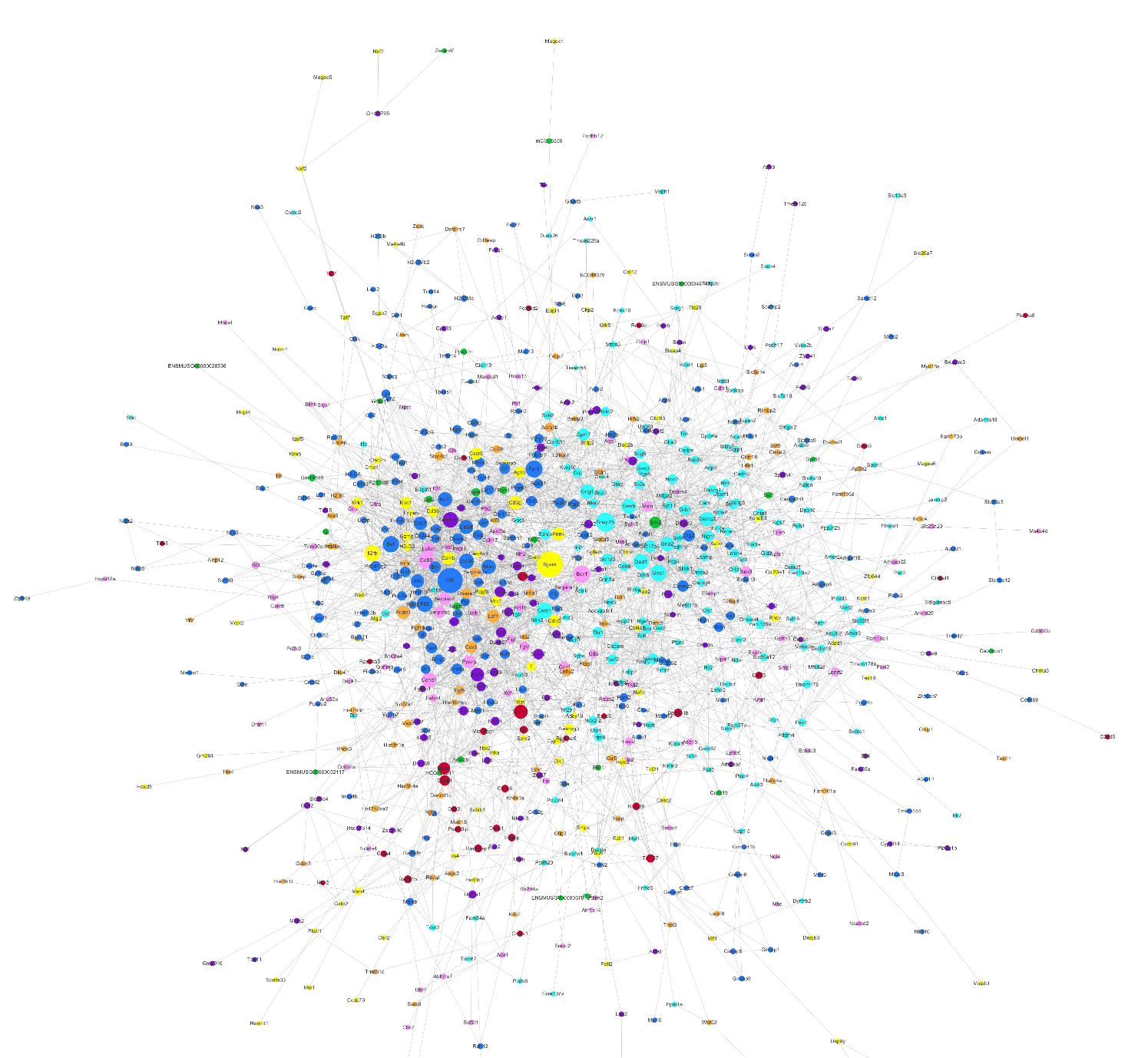


**Supplementary Figure 3.** A continued protein protein interaction network that was bridged by 25 other genes. The continued protein-protein interaction network contains 823 nodes and 3113 edges.


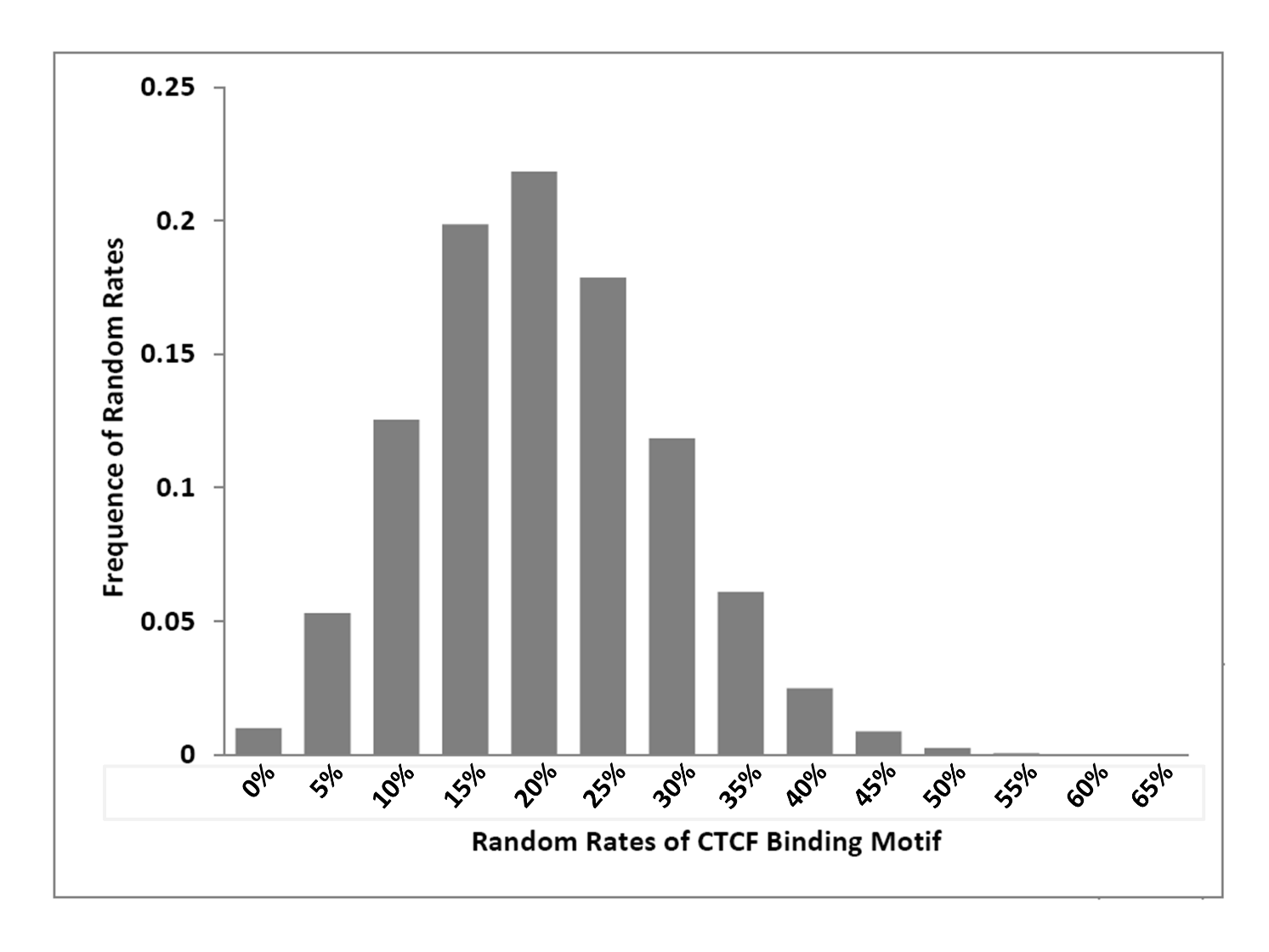


**Supplementary Figure 4.** Random occupancy rates of the genome-wide CTCF binding sites.


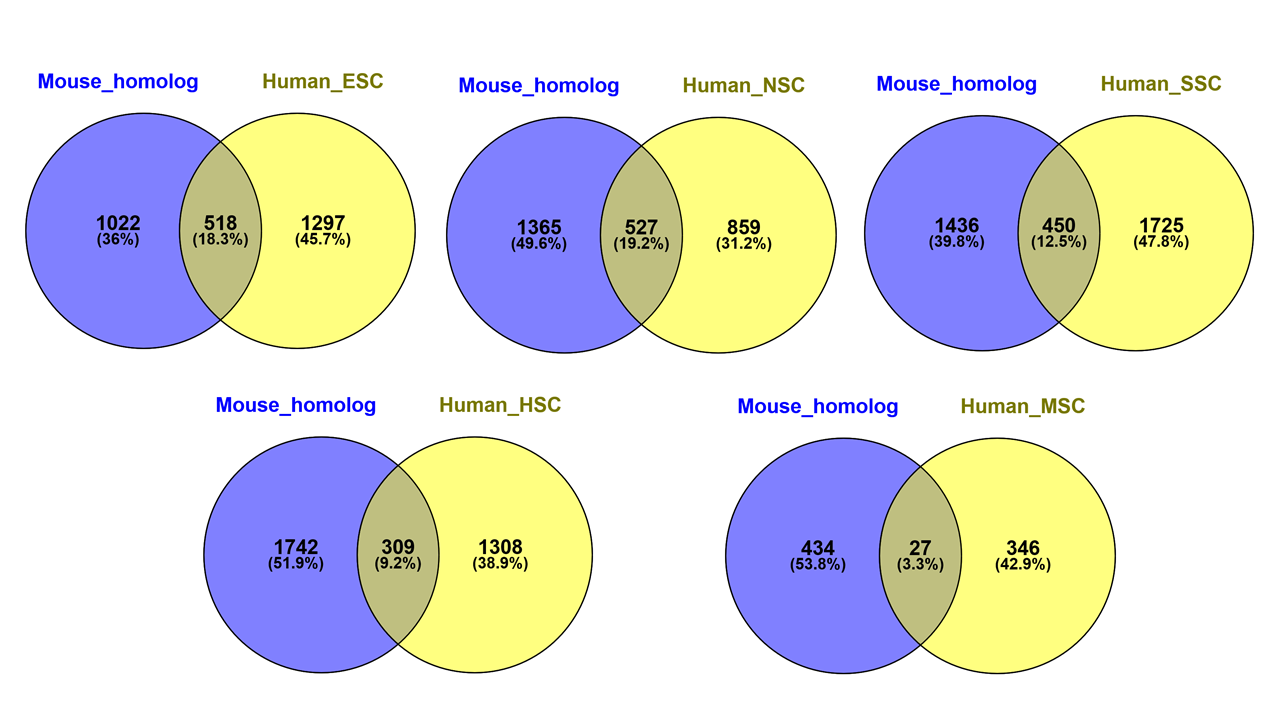


**Figure S5. The percentages of common up-regulated genes both in mouse and human stem cells are different among the 5 types of human stem cells.** Mouse_homolog means the up-regulated genes of mouse respective stem cells corresponding to human homologous genes. The percentage was calculated by compared gene amount of respective sections to the sum of mouse and human up-regulated genes.

**Supplementary Tables**

Supplementary Table S1. Primer sequences used in this study.

Supplementary Table S2. The highly expressed gene lists of each types of stem cell.

Supplementary Table S3. The gene lists of stem cells respective special modules. Those identified mouse stem cell special genes which also up-regulated in human stem cells were highlighted with yellow color.

Supplementary Table S4. Whether or not of those core genes were up-regulated in the respective human stem cells.

Supplementary Table S5. The relative expression foldchanges of those annotated homologous genes between mouse and human.
